# Decision‐making regarding primary prevention implantable cardioverter‐defibrillators among older adults

**DOI:** 10.1002/clc.23315

**Published:** 2019-12-23

**Authors:** Paul L. Hess, Daniel D. Matlock, Sana M. Al‐Khatib

**Affiliations:** ^1^ Rocky Mountain Regional VA Medical Center Aurora Colorado; ^2^ Cardiology Section University of Colorado Anschutz Medical Campus Aurora Colorado; ^3^ Duke Clinical Research Institute Duke University Medical Center Durham North Carolina

**Keywords:** decision‐making, implantable cardioverter defibrillator, older adults

## Abstract

Most implantable cardioverter defibrillators (ICDs) are implanted for the purpose of primary prevention of sudden cardiac death among older patients with heart failure with reduced ejection fraction. Shared decision‐making prior to device implantation is guideline‐recommended and payer‐mandated. This article summarizes patient and provider attitudes toward device placement, device efficacy and effectiveness, potential periprocedural complications, long‐term events such as shocks, quality of life, costs, and shared decision‐making principles and recommendations. Most patients eligible for an ICD anticipate more than 10 years of survival. Physicians are less likely to offer an ICD to patients ≥80 years of age given a perceived lack of benefit. There is a dearth of data from randomized clinical trials addressing device efficacy among older patients; there is a need for more research in this area. However, currently available data support the use of ICDs irrespective of age provided life expectancy exceeds 1 year. Advanced age is independently associated with complications at the time of device placement but not the risk of device infection. The risk of inappropriate shock may be comparable or lower than that of younger patients. While quality of life is generally not adversely impacted by an ICD, a subset of patients experience post‐traumatic stress disorder. ICDs are cost‐effective from societal and health care sector perspectives; however, out‐of‐pocket costs vary according to insurance type and level. Shared decision‐making encounters may be incremental and iterative in nature. Providers are encouraged to partner with their patients, providing them counsel tailored to their values, preferences, and clinical presentation inclusive of age.

## 1 INTRODUCTION

Sudden cardiac death is a significant public health hazard, accounting for 230 000 to 350 000 deaths annually in the United States over the past 20 to 30 years.^1^ The implantable cardioverter defibrillator (ICD) is the mainstay of therapy for patients at an elevated risk of sudden death. More than 200 000 ICDs are implanted annually, the majority of which are for a primary prevention indication among patients with heart failure with reduced ejection fraction.^2^ The average age of patients receiving an ICD in the United Stated is 67 years.^3^ For the purposes of the current manuscript, “advanced” or “older” age is generally defined as chronologic age ≥65 years. Biologic age may nonetheless diverge from chronologic age; some individuals 85 years of age may have biology similar to that of individuals 65 years of age and vice versa. Given the number of Americans ≥65 years of age is expected to increase from 46 million in 2014 to 74 million in 2030,^4^ the number of older patients eligible for ICDs may correspondingly increase over time. Nonetheless, older patients have historically been less likely to receive an ICD than their younger counterparts (odds ratio [OR] 0.89, 95% confidence interval [CI] 0.87‐0.91 per 5‐year increase in age).^5^ Low rates of ICD use among older patients are likely multifactorial and in part related to the lower likelihood of clinicians counseling their patients about ICDs, which decreases with advancing patient age >80 years (OR 0.90, 95% CI 0.89‐0.92, per 1‐year increase ≥80 years).^6^

Current professional guidelines indicate that clinicians should engage eligible patients in shared decision‐making about ICDs.^1^ Specifically, device effectiveness, safety, and potential complications should be discussed in light of patients' health goals, preferences, and values. Notably, advanced age is not an exclusion criterion for ICDs. Indeed, the 2017 guideline provided the following recommendation regarding older patients that was based on the results of an independently conducted systematic review: “For older patients and those with significant comorbidities, who meet indications for a primary prevention ICD, an ICD is reasonable if meaningful survival of greater than 1 year is expected.”^1^ However, this recommendation was based on observational data, and more data on the efficacy and safety of ICDs, preferably from randomized clinical trials, are needed in older patients. In 2018, the Centers for Medicare and Medicaid Services began to require that patients participate in shared decision‐making as a condition for reimbursement for primary prevention ICDs.^7^ To assist clinicians preparing for guideline‐indicated and payer‐mandated shared decision‐making with older patients considering a primary prevention ICD, the intent of this article is to summarize patient and provider attitudes toward device placement, device efficacy and effectiveness, periprocedural complications, long‐term events such as shocks and generator replacement, as well as additional considerations such as quality of life and costs. We then provide direction on how to best guide older adults toward a value‐concordant decision regarding implantation of primary prevention ICDs.

## 2 PATIENT AND PROVIDER ATTITUDES TOWARD DEVICE IMPLANTATION

Several key features of patients' perspectives toward device placement have been described. Despite having symptomatic heart failure, most patients eligible for an ICD anticipate more than 10 years of survival.^8^ Many older patients who go on to receive an ICD may express a clear desire to prolong their lives and the importance of ICDs in that effort. They may do so without considering competing health risks unrelated to sudden death.^9^ Some patients prefer to focus on their current health status rather than its future trajectory. Asymptomatic patients with satisfaction regarding their quality of life may in fact choose against ICD implantation, noting they will reconsider it if their symptoms worsen in the future.^10^ These patients should be educated about the fact that in the majority of patients, sudden cardiac death occurs in the absence of symptoms. Others may favor not placing an ICD given a negative body image or perceptions regarding potential lifestyle changes such as exercise or sex.^11^ Importantly, patients' preferences regarding how involved they wish to be in the decision to receive an ICD span the full spectrum. While some wish to abdicate the decision entirely to their physicians, others desire to play a more active role.^12^

Studies on physicians' attitudes toward device implantation are sparse. Available data indicate cardiologists are generally more aware of guideline indications for device implantation than primary care physicians,^13^ leading to a potential gap in referral for device consideration for some eligible patients. In general, physicians are less likely to offer an ICD to patients ≥80 years of age^14^ given a perceived lack of benefit.^13^ The role of shared‐decision‐making especially in such patients could not be overemphasized.

## 3 EFFICACY AND EFFECTIVENESS

Landmark clinical trials demonstrated ICD efficacy in the reduction of mortality among patients with LV systolic dysfunction. Although older age was not an exclusion criterion, older patients were underrepresented in these trials. Consequently, several meta‐analyses of the randomized data were performed (Table 1).^15^, ^16^, ^17^ In one meta‐analysis, the reduction in mortality among patients older than 60 years of age was significant (hazard ratio (HR) 0.75, 95% CI 0.61‐0.91) but less pronounced compared with younger patients (HR 0.65, 95% CI 0.50‐0.83).^15^ A second meta‐analysis yielded similar findings among patients ≥65 years of age (HR 0.66, 95% CI 0.50‐0.87). Among patients ≥75 years of age, the survival benefit remained but was attenuated (HR 0.73, 95% CI 0.51‐0.97).^16^ These analyses were performed using trial‐level estimates, precluding the possibility of adjustment for differences in comorbidities and medical therapies as well as the evaluation of age in a continuous rather than categorical fashion. Thus, a patient‐level analysis of the clinical trials was performed. In unadjusted analyses, ICD recipients were less likely to die than nonrecipients in all age groups. In adjusted analyses, point estimates indicated ICD efficacy persisted but became less pronounced with increasing age. The sample size of patients aged ≥75 years was limited (*n* = 390), leading to continued uncertainty regarding the survival benefit of ICDs in this age subgroup.^17^

**Table 1:** Meta‐analyses of the influence of age on ICD efficacy

Meta‐analysis	Trials	Age group	ICD effect	
Santangeli et al^15^	MADIT‐II	Younger patients	HR 0.65 (95% CI 0.50‐0.83)	
	DEFINITE	<60 y in MADIT‐II		
	SCD‐HeFT	<65 y in DEFINITE and SCD‐HeFT		
		Older patients	HR 0.75 (0.61‐0.91)	
		≥65 y in MADIT‐II		
		≥60 y in DEFINITE and SCD‐HeFT		
Kong et al^16^	MADIT‐I	≥65 y^a^	HR 0.66, 95% CI 0.50‐0.87	
	MUSTT	≥75 y^b^	HR 0.73, 95% CI 0.51‐0.97	
	MADIT‐II			
	DEFINITE			
	SCD‐HeFT			
Hess et al^17^	MADIT‐I	<55 y	HR 0.48, 95% PCI 0.33‐0.69	
	MUSTT	65‐74 y	HR 0.69, PCI 0.53‐0.90	
	MADIT‐II	≥75 y	HR 0.54, 95% PCI 0.37‐0.78	
	DEFINITE			
	SCD‐HeFT			

Abbreviations: CI, confidence interval; DEFINITE, the Defibrillators in Nonischemic Cardiomyopathy Treatment; HR, hazard ratio; ICD, implantable cardioverter defibrillator; MADIT, the Multicenter Automatic Defibrillator Implantation Trial; MUSTT, the Multicenter UnSustained Tachycardia Trial; PCI, posterior credible interval; SCD‐HeFT, the Sudden Cardiac Death in Heart Failure Trial.

a Excluded MUSTT.

b Excluded MADIT‐I.

The Danish Study to Assess the Efficacy of ICDs in Patients with Non‐Ischemic Systolic Heart Failure on Mortality (DANISH) showed the ICD was not efficacious among patients with nonischemic cardiomyopathy (HR 0.87, 95% CI 0.68‐1.12).^18^ However, when these data were combined with all randomized trials that included patients with nonischemic cardiomyopathy, there was a survival benefit with primary prevention ICDs (HR 0.75, 95% CI 0.61‐0.93).^19^ These disparate findings have been attributed to the large proportion of patients in the DANISH study receiving cardiac resynchronization therapy, the high use of effective medical therapy, and requiring an elevated pro‐BNP level for inclusion in the trial. In this setting, a secondary analysis of the DANISH study demonstrated a linearly decreasing relationship between ICD and mortality with increasing age (HR 1.03, 95% CI 1.00‐1.06). Among patients >70 years of age, a survival benefit of ICD placement was not observed (HR 1.05, 95% CI 0.68‐1.62).^20^ Potential reasons underlying this finding include the aforementioned aspects of the DANISH study as well as a limited sample size of older patients.

Randomized trials have shown that cardiac resynchronization therapy with an ICD is superior to an ICD alone in the reduction of mortality and heart failure exacerbations among eligible patients with left ventricular systolic dysfunction and a widened QRS complex.^21^, ^22^, ^23^ Similar to the primary prevention ICD trials, older age was not an exclusion criterion. While meta‐analyses of the trials do not report the association of age with device efficacy, subgroups by age were reported in all of the trials. These unadjusted analyses did not demonstrate a reduction in efficacy with increasing age. In a secondary analysis of MADIT‐CRT, the risk of death or hospitalization or hospitalization with heart failure was reduced among patients ≥75 years of age (*n* = 331, HR 0.57, 95% CI 0.37‐0.87) and 60 to 74 years of age (*n* = 941, HR 0.55, 0.41‐0.72) but not among those <60 years of age (*n* = 548, HR 0.80, 95% CI 0.52‐1.23).^24^ These findings were driven by a reduction in rehospitalization more so than mortality.^24^ Similar results were observed in the Comparison of Medical Therapy, Pacing, and Defibrillation in Heart Failure trial.^21^ More data are needed on CRT‐D vs CRT‐P, especially in older patients. A pilot study funded by the National Institutes of Health is currently underway to compare these two therapies in patients 75 years of age and older.^25^ If proven feasible, a larger trial with adequate statistical power to compare survival will follow.

Randomized trials often enroll patients with fewer comorbidities than those seen in general clinical practice. Moreover, they are typically conducted in highly monitored and controlled settings. Thus, whether the randomized data apply to real‐world clinical practice has been an area of keen interest. Rigorous analyses comparing registry data to trial data have shown that patients eligible for ICD therapy based on trial criteria and yet who are seen in routine clinical practice have better survival with an ICD. Specifically, there was no difference in survival between ICD recipients in the registry and the trials whether patients were comparable to those enrolled in MADIT‐II (HR 1.06, 95% CI 0.85‐1.31) or SCD‐HeFT (HR 1.16, 95% CI 0.97‐1.38).^26^ The median ages of MADIT‐II‐like and SCD‐HeFT‐like patients were 68 and 67 years, respectively. In a secondary analysis of patients 65 years and older, the findings were comparable to the primary results. These data generally support the use of ICDs in routine clinical care. However, there was a dearth of data on patients >80 years of age, limiting the degree to which the findings can be extrapolated to patients in this advanced age group.

Observational analyses of real‐world datasets to understand the role of age in ICD real‐world effectiveness have been undertaken. In a cohort of 965 patients with ischemic or nonischemic cardiomyopathy, ICD therapy was shown to be associated with a lower risk of death (0.69, 95% CI 0.50‐0.96). This relationship was consistent after stratification by age (<65 years (*n* = 283); 65 to 74 years (*n* = 313); ≥75 years (*n* = 269).^27^ Similarly, in the American Heart Association Get With the Guidelines‐Heart Failure registry linked with Medicare claims, ICD therapy was associated with a lower risk of death over 3 years after device placement up to 84 years of age.^28^ In a meta‐analysis of predominantly observational data, a survival advantage of ICD vs no ICD was seen among older patients (HR 0.75, 95% CI 0.67‐0.83).^29^ In the Swedish Heart Failure Registry, compared with ICD nonreceipt, ICD receipt was associated with a lower risk of death within 1 year (HR 0.73, 95% CI 0.60‐0.90 and 5 years (HR 0.88, 95% CI 0.78‐0.99). Results were consistent across age subgroups of age < 75 vs ≥75 years (1‐year interaction *P* = .30, 5‐year interaction *P* = .87).

Observational studies to understand the role of age among recipients of cardiac resynchronization therapy with ICD vs ICD alone among eligible patients have also been performed. In the National Cardiovascular Data Registry's ICD Registry linked with Medicare claims, cardiac resynchronization therapy was associated with a lower risk of mortality (HR 0.82, 95% CI 0.73‐0.93).^30^ Using the same registry linked with the national death index, cardiac resynchronization therapy was associated with better 1‐year survival than an ICD alone (82.1% vs 77.1%). A differential effect by age was not observed (age groups <55 years, 55‐64 years, 65‐74 years, 75‐84 years, ≥ 85 years; interaction *P* = .86).^31^

In addition to age, medical comorbidities, which are more prevalent with increasing age, influence the likelihood of patient survival irrespective of ICD placement. Statistical models predicting short‐ and long‐term survival related to ICD implantation using data from either trials^32^, ^33^ or observational datasets^34^, ^35^ have been developed. These models show that a number of comorbidities influence patient survival, including atrial fibrillation, chronic kidney disease, chronic obstructive pulmonary disease, and diabetes mellitus (Table 2). Among these, chronic kidney disease has particularly been shown to have a big influence.^35^ The risk of death after primary prevention ICD placement is in fact proportional to the severity of chronic kidney disease.^36^ It is most pronounced among patients with end‐stage renal disease on dialysis; a total of 22.5% of ICD recipients on dialysis die within 1 year of device implantation.^37^ Notably, the acuity of illness at the time of device placement may play a role in overall device effectiveness; patients hospitalized with heart failure or other comorbidities may not derive as much survival benefit as those receiving the device in an elective setting.^38^

**Table 2:** Comorbidities associated with reduced ICD effectiveness

Atrial fibrillation^33^, ^34^
Blood urea nitrogen^33^
Chronic kidney disease^34^
Chronic lung disease^35^
Chronic obstructive pulmonary disease^34^
Dementia^39^
Diabetes mellitus^34^
Dialysis^35^
Frailty^39^
Left ventricular ejection fraction <20%^34^
New York Heart Association class^32^, ^33^, ^34^
QRS duration^33^
Systolic blood pressure < 120 mm Hg^35^

Abbreviation: ICD, implantable cardioverter defibrillator.

In a cohort derived from the National Cardiovascular Data Registry's ICD Registry linked with Medicare claims, geriatric conditions such as frailty and dementia were present in more than 10% of patients ≥65 years of age receiving an ICD and were associated with more than twice the risk of death within 1 year of device implantation (22% among patients with frailty, 27% among patients with dementia, 12% in the overall cohort).^39^ Multimorbid patients, including those with chronic obstructive pulmonary disease and diabetes in conjunction with frailty and/or dementia, had still worse 1‐year death rates (dementia with frailty, 29%; frailty with chronic obstructive pulmonary disease, 25%; frailty with diabetes, 23%).^39^ In part a consequence of the substantial comorbidity burden and the concomitant risk for nonsudden death, one in two patients receiving an ICD after the age of 65 either die or are admitted to a hospice facility within 5 years of device receipt. Factors associated with reduced time to hospice enrollment include advanced age, heart failure class, and an ejection fraction <20%.^40^ These data suggest that there may be opportunities to integrate palliative care principles into the decision to implant an ICD and to seek specialized palliative consultation even if life‐prolonging therapies such as ICDs are desired.^40^

While prior randomized and observational studies can inform current clinical care, several ongoing studies have the potential to shape our understanding of ICD efficacy and effectiveness moving forward. To address our gap in knowledge regarding ICD efficacy in older patients, a multisite, randomized clinical trial comparing ICD implantation and optimal medical therapy to optimal medical therapy alone among patients 70 years of age and older is underway in the Department of Veterans Affairs health care system.^41^ To address our knowledge gap regarding ICD effectiveness in the modern therapeutic era, a multisite, observational cohort study is underway in 44 centers across 15 European Union countries.^42^ While the latter study does not specifically target older patients, it may nonetheless yield updated information on the effectiveness of primary prevention ICDs among patients of advanced age given an anticipated enrollment exceeding 2300 patients.

## 4 PERIPROCEDURAL COMPLICATIONS AND LONG‐TERM EVENTS

The National Cardiovascular Data Registry's ICD Registry has been used to examine the prevalence of in‐hospital, perioperative events. A total of 1.8% of patients experienced at least one in‐hospital adverse complication. The most common adverse events were lead dislodgement (0.66%), hematoma (0.30%), death (0.26%), pneumothorax (0.24%), and cardiac arrest (0.20%). Additional adverse outcomes included coronary venous dissection, device‐related infection, pericardial tamponade, cardiac perforation, stroke, hemothorax, and a set screw problem.^3^ In a risk score developed for in‐hospital adverse events, a total of 13 risk factors were independently associated with adverse outcomes. Age was a significant variable in the model (HR 1.09, 95% CI 1.05‐1.13 per 10‐year increase). Importantly, a number of comorbidities also influenced the risk of complications, including prior percutaneous coronary intervention, the absence of prior coronary artery bypass grafting, cerebrovascular disease, diabetes, and dialysis.^3^ The number of device leads also influenced the risk of complications (Table 3).^3^ While some complications are unavoidable, others may be evaded by careful patient selection and optimized device choices.

**Table 3:** Factors associated with in‐hospital complications after ICD implantation^3^

Abnormal electrical conduction
Age
Blood urea nitrogen
Cardiac arrest
Cardiac rhythm
Cerebrovascular disease
Chronic lung disease
Diabetes mellitus
Dialysis
Female sex
Glomerular filtration rate
Hemoglobin
New York Heart Association class
No prior CABG
Nonischemic dilated cardiomyopathy
Number of leads
Procedure type
Prior PCI
Reason for admission
Sodium
Systolic blood pressure

Abbreviations: CABG, coronary artery bypass grafting; ICD, implantable cardioverter defibrillator; PCI, percutaneous coronary intervention.

Among patients ≥65 years of age receiving either an initial implant or generator change, 1.7% of patients develop an ICD infection within 6 months after discharge.^43^ These are viewed as particularly problematic >1 year after device placement, as they frequently result in device extraction with its attendant risks, including sternotomy and/or death.^44^, ^45^ Age per se does not appear to impact the risk of infection. By contrast, a number of comorbidities, including prior valvular surgery, chronic lung disease, cerebrovascular disease, dialysis, warfarin use, chronic immunosuppression inclusive of steroid‐sparing and steroid agents, as well as procedural factors such as whether or not the patient has received a prior device, are significant factors in this regard (Table 4).^43^, ^46^ Though not specifically examined, factors related to immunosuppression such as a high load of human immunodeficiency virus likely also play a role. Given that the incidence of many of these comorbidities increases with age, age is indirectly associated with increased risk of infection.

**Table 4:** Factors associated with ICD infection

Cerebrovascular disease^43^
Chronic lung disease^43^
Chronic immunosuppression^46^
Dialysis^43^, ^46^
Other adverse events^43^
Prior infection^46^
Prior valvular surgery^43^
Reimplantation^43^
Warfarin use^43^

Abbreviation: ICD, implantable cardioverter defibrillator.

ICD shocks may be classified as appropriate or inappropriate. The former are triggered by a life‐threatening arrhythmia while the latter are not. Observational studies have elucidated rates of shock therapies and evaluated the potential for differences in shock risk across age groups. In the National Cardiovascular Data Registry ICD Registry linked with medical record and device therapy data, the 3‐year risk of shock was 36% (appropriate, 24%; inappropriate, 12%). Inappropriate therapy was less common among patients ≥65 years compared with those <65 years (adjusted HR 0.72, 95% CI 0.54‐0.95).^47^ The presence of atrial fibrillation is associated with a higher risk of inappropriate therapy (HR 2.20, 95% CI 1.68‐2.87)^47^ as well as reduced ICD effectiveness.^34^ By contrast, the Ontario ICD Database showed that the risk for appropriate shocks was similar across age groups (reference, patients 18‐49 years; HR 0.83, 95% CI 0.54‐1.29 among patients 50‐59 years; HR 0.77, 95% CI 0.50‐1.18 among patients 60‐69 years; HR 0.68, 95% CI 0.44‐1.07 among patients 70‐79 years; and HR 0.71, 95% CI 0.38‐1.34 among patients ≥80 years; *P* for trend across age groups = .130). As seen in other studies, patients ≥80 years were underrepresented.^48^ An additional observational study based in Switzerland and Austria similarly found that age (age < 75 years vs age ≥ 75 years) was not associated with the risk of appropriate ICD shocks.^36^ It is important to note that with the implementation of key device programming parameters, a significant proportion of inappropriate shocks may be avoided.^49^

After initial ICD placement, the device may require replacement when the battery reaches the end of life. At the time of the procedure, the device can be replaced and the leads are generally left in place. At the time of device replacement, patients are often older compared with those receiving an initial implant (70.7 vs 67.5 years of age).^50^ After device replacement, 1‐, 3‐, and 5‐year mortality is 9.8%, 27.0%, and 41.2%, respectively.^51^ Older age is associated with a higher risk of mortality (HR 1.43, 95% CI 1.41‐1.45). Cardiovascular comorbid conditions such as atrial fibrillation and heart failure as well as noncardiovascular conditions such as chronic lung disease, cerebrovascular disease, diabetes mellitus, and kidney disease are also associated with poorer survival. In the absence of a comparison group, the risk reduction in mortality associated with device replacement is not yet known.^51^

## 5 ADDITIONAL CONSIDERATIONS

Quality of life studies were conducted in the primary prevention clinical trials. The body of data indicates there is no clear evidence of a reduction in quality of life associated with ICD placement.^52^ Nonetheless, a subset of patients who experience an ICD shock develop post‐traumatic stress disorder, which in turn is associated with significant adverse outcomes inclusive of premature death.^53^ While ICDs alone have not been associated with a reduction in quality of life, patient's quality of life, health, and/or functional status may nonetheless deteriorate as a consequence of the inexorable progression of heart failure or other comorbidity. Patients' perspective on the risk/benefit ratio of ICD therapy may in turn evolve over time such that what made sense at the time of implantation may make less sense with advancing age. Thus, while patients may decide to proceed with device implantation, they may decide to forego the potential survival benefit at the time of generator change or deactivate the device at some time in the future after device placement.

In cost‐effectiveness analyses, an outcome of central interest is survival. Assessments are contingent upon the perspective taken, whether it be that of society at large, the health care sector, or individual patients. From the perspective of the health care sector, analyses of the primary prevention ICD trials reported increased survival with higher lifetime costs of medical care compared with the absence of an ICD.^54^, ^55^, ^56^ Incremental cost‐effectiveness ratios were typically less than $50 000 per year of life added, falling within an acceptable range supported by cardiovascular professional guidelines.^57^ Nonetheless, patients may be interested in outcomes in addition to survival such as quality of life (described above) and may also express interest in out‐of‐pocket costs. Most costs are incurred at the time of device implantation. Routine postimplant care will often involve an in‐person follow‐up clinic visit within 1 to 3 months of device implantation and annual in‐person clinic visits with the rest of follow‐up occurring through remote device interrogation. Additional costs may accrue in the event of a procedural complication, device infection, or ICD shock. Travel time and time off work may also play an important role for many patients and their caregivers. The magnitude of out‐of‐pocket costs depends on patients' type and level of insurance coverage.

## 6 SHARED DECISION‐MAKING PRINCIPLES AND IMPLEMENTATION

**Figure 1:**
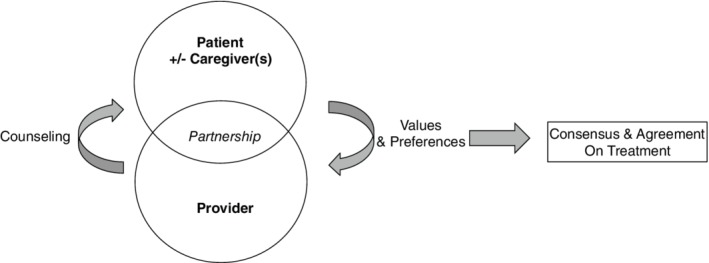
The shared decision‐making theoretical framework highlights key elements of shared decision‐making: (a) involvement of at least the patient and provider, (b) meaningful sharing of information, and (c) consensus building and agreement on the treatment to implement

The National Academy of Medicine defined patient‐centered care as “care that is respectful and responsive to individual patient preferences, needs, and values” and ensures that “patient values guide all clinical decisions.”^58^ Shared decision‐making helps patients to understand their options as well as downstream results of their decisions. Their values and preferences are elicited. Clinicians in turn take on a role of advisor or partner rather than a paternalistic arbiter.^59^ This theoretical framework encompasses and supersedes the legalities of informed consent by aligning medical therapies with patient preferences and values (Figure 1).

At the outset of shared decision‐making, it may be helpful to characterize patients' desired level of engagement in the decision‐making process. Some patients may prefer that physicians make the decision for them; others may wish to make the decision with little or no physician input. Our experience has been that most patients lie somewhere along the spectrum of these extremes. Among patients who decide to partner with their provider in the selection of treatment options, assessing the level of detail desired in the discussion is an additional important and at times iterative step. Select patients may wish to understand precise values and percentages. In such instances, the detailed discussion above may prove particularly useful. Others may prefer more general descriptions, e.g. “gisting.”^60^ In this case, the above discussion may provide the substrate for conveying central concepts. Understanding patients' goals and values is an essential component to the shared decision‐making process. Patient goals and preferences should be informed by their biologic more than their chronologic age as well as burden of comorbid illnesses, including the presence or absence of chronic diseases such as kidney disease or malignancies. Clinicians are encouraged to integrate the factors contributing to patients' prognosis and convey their significance in a readily digestible manner. Patients' general characteristics as a medical maximizer or minimizer may guide discussions; some emerging research suggests that some individuals tend toward seeking medical care in most instances, while others may prefer to avoid medical intervention in most cases.^61^ These general health behaviors may then engender a more tailored discussion about an individual patient's interest in ICD placement. Finally, deciding whether to engage family members and caretakers in discussions is critical, particularly for older adults with cognitive impairments. Having set the stage for a fruitful interaction, providers and patients may then walk through risks, benefits, and alternatives of ICD placement. Included in these discussions should be notions that decisions may be incremental, iterative, and/or revisited. After the initial discussion, patients may wish to reflect on their values and discuss their implications with family members or loved ones. Providers may then reengage in the discussion later. Patients' values and preferences may in fact be dynamic and evolve over time and with advancing age. Even after device implantation, discussions may continue to take place and the device may in turn be deactivated per patient preferences.

In a Cochrane review of 105 studies, compared with usual care, decision aids have been shown to better characterize patient values and increase patient's knowledge base, leading to more informed, value‐concordant decisions.^62^ They may use a variety of media formats, including interactive websites, booklets, or videos. While there is a solid evidence base for decision aids in general, their uptake into routine clinical practice has generally been lackluster. Addressing the issue of real‐world implementation in 2018, the Centers for Medicare and Medicaid mandated their use prior to ICD implantation in the national coverage determination.^7^ In the memo, use of a decision aid developed by the Colorado Program for Patient‐Centered Decisions was encouraged.^63^ Clinicians may use this or another evidence‐based decision aids before, during, or after clinic visits to stimulate and guide provider‐patient discussions. Unfortunately, the Centers for Medicare and Medicaid did not offer a clear definition of what is meant by an evidence‐based decision aid. Whether an ICD decision aid can improve decisional quality and be better tailored to sex‐ or race/ethnicity‐based subgroups remain areas of active research.^64^, ^65^ While the decision aid is intended for general use by clinicians and patients considering primary prevention ICD placement, the discussion should be tailored to individual patients, specifically accounting for their age as it relates to their risk of sudden vs nonsudden death as informed by their comorbidity burden; device effectiveness, safety, periprocedural complications; long‐term events such as shocks, and generator replacement; and additional considerations such as quality of life and costs. These issues should be addressed within the context of individual patient preferences and values.

## 7 CONCLUSIONS

There is a paucity of data regarding ICD efficacy among older patients. The balance of available data, whether they be pooled from randomized trials or derived from observational datasets, supports the use of ICDs irrespective of age provided life expectancy exceeds 1 year. Advanced age has been shown to be an independent risk factor associated with complications at the time of ICD implantation; however, advanced age per se is not associated with increased risk of device infection. Rather, its impact may be indirect via comorbidities that are prevalent with advanced age. Compared with younger patients, older patients are as or less likely to receive an inappropriate shock. Advanced age as well as comorbidities are independently associated with increased mortality at the time of device replacement. ICDs do not have a significant impact on most patients' quality of life; nonetheless, a subset of patients will experience post‐traumatic stress disorder. While ICDs have been shown to be cost‐effective from societal and health care sector perspectives, some patients may be more interested in out‐of‐pocket costs, which vary by level and type of insurance coverage.

Shared decision‐making at the time of device placement is recommended by professional guidelines and is now mandated by the Centers for Medicare and Medicaid Services as a criterion for reimbursement. During shared decision‐making encounters, providers should elicit patients' values and preferences and counsel patients accordingly, accounting for not only their age but also comorbidities and life expectancy. An ICD decision aid may inform patient‐provider interactions, which may be incremental and iterative in nature.
